# Hypertrophic Preconditioning Attenuates Myocardial Ischaemia‐Reperfusion Injury by Modulating SIRT3‐SOD2‐mROS‐Dependent Autophagy

**DOI:** 10.1111/cpr.13051

**Published:** 2021-05-11

**Authors:** Lei‐Lei Ma, Fei‐Juan Kong, Zheng Dong, Kai‐Yue Xin, Xing‐Xu Wang, Ai‐Jun Sun, Yun‐Zeng Zou, Jun‐Bo Ge

**Affiliations:** ^1^ Department of Cardiology Shanghai Institute of Cardiovascular Diseases Zhongshan Hospital Fudan University Shanghai China; ^2^ NHC Key Laboratory of Viral Heart Diseases Shanghai China; ^3^ Key Laboratory of Viral Heart Diseases Chinese Academy of Medical Sciences Shanghai China; ^4^ Department of Endocrinology and Metabolism Shanghai General Hospital Shanghai Jiao Tong University School of Medicine Shanghai China; ^5^ Department of Cardiology Cheeloo College of Medicine Shandong University Jinan China

**Keywords:** autophagy, hypertrophic preconditioning, ischaemia‐reperfusion injury, SIRT3

## Abstract

**Background:**

Ischaemic preconditioning elicited by brief periods of coronary occlusion and reperfusion protects the heart from a subsequent prolonged ischaemic insult. Here, we test the hypothesis that short‐term non‐ischaemic stimulation of hypertrophy renders the heart resistant to subsequent ischaemic injury.

**Methods and Results:**

Transient transverse aortic constriction (TAC) was performed for 3 days in mice and then withdrawn for 4 days by aortic debanding, followed by subsequent exposure to myocardial ischaemia‐reperfusion (I/R) injury. Following I/R injury, myocardial infarct size and apoptosis were significantly decreased, and cardiac dysfunction was markedly improved in the TAC preconditioning group compared with the control group. Mechanistically, TAC preconditioning markedly suppressed I/R‐induced autophagy and preserved autophagic flux by deacetylating SOD2 via a SIRT3‐dependent mechanism. Moreover, treatment with an adenovirus encoding SIRT3 partially mimicked the effects of hypertrophic preconditioning, whereas genetic ablation of SIRT3 in mice blocked the cardioprotective effects of hypertrophic preconditioning. Furthermore, in vivo lentiviral‐mediated knockdown of Beclin 1 in the myocardium ameliorated the I/R‐induced impairment of autophagic flux and was associated with a reduction in cell death, whereas treatment with a lentivirus encoding Beclin 1 abolished the cardioprotective effect of TAC preconditioning.

**Conclusions:**

The present study identifies TAC preconditioning as a novel strategy for induction of an endogenous self‐defensive and cardioprotective mechanism against cardiac injury. Specifically, TAC preconditioning reduced myocardial autophagic cell death in a SIRT3/SOD2 pathway‐dependent manner.

## Graphical abstract

Hypertrophic preconditioning exerted marked protective effects by eliminating mitochondrial‐derived superoxide and suppressing autophagic cell death. SIRT3 partially mimicked the effects of hypertrophic preconditioning, whereas genetic ablation of SIRT3 in mice blocked the cardioprotective effects of hypertrophic preconditioning.

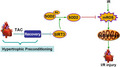

## 1 INTRODUCTION

Acute myocardial infarction (AMI) remains prevalent throughout the world and is commonly caused by thrombotic occlusion of the coronary artery, resulting in cardiomyocyte death, reparative fibrotic healing, left ventricular remodelling and ultimately heart failure.^1^ Despite progress in reperfusion therapy, nearly 10% of AMI subjects die during index hospitalization, and 25% of survivors develop chronic heart failure.^2^ One explanation for these poor outcomes is that reperfusion therapy, such as thrombolytic agents and primary percutaneous coronary intervention, does not rescue dead cardiomyocytes or ameliorate the deterioration of myocardial function.^3^, ^4^ Myocardial ischaemia‐induced DNA damage and mitochondrial reactive oxygen species (mROS) overload are the primary contributors to cardiomyocyte death.^5^ It has been suggested that autophagy plays a pivotal role in cardiomyocyte death and cardiac remodelling to maintain cardiac function and mitochondrial homeostasis in the heart.^6^, ^7^ Recently, we reported that autophagy dysfunction leads to mitochondrial loss, oxidative injury, and cell death in ischaemia‐reperfusion (I/R)‐induced damage, and therapeutic strategies targeting autophagy ameliorated I/R injury.^8^ These findings highlight the vital role of autophagy in I/R‐induced cardiac injury and suggest that modulating autophagy is a viable and novel therapeutic strategy.

Myocardial ischaemic preconditioning (IPC) induced by brief episodes of ischaemia followed by reperfusion protects the heart from a subsequent prolonged ischaemic challenge.^9^ Given the clinical implications, intense research has been conducted to dissect the molecular mechanisms underlying the cardioprotective effects of IPC. In contrast, non‐ischaemic preconditioning has received relatively little consideration and remains largely underexplored. Preconditioning with mechanical stretch, heat stress and pharmacological interventions can mimic the cardioprotective effects of IPC.^10^, ^11^ It was noted that intermittent pressure overload promoted improved myocardial performance and induced a moderate hypertrophic response, as well as a favourable gene expression profile.^12^, ^13^ Moreover, angiotensin II or norepinephrine preconditioning was shown to promote protein kinase C activation and restrict myocardial infarction in isolated perfused rabbit hearts,^14^, ^15^ and transient left ventricular pressure overload decreased cardiomyocyte apoptosis in rats exposed to I/R.^16^ Recently, the reduction in left ventricular preload by percutaneous left atrial‐to‐femoral artery bypass inhibited myocardial infarction and activated the reperfusion injury salvage kinase pathway.^17^, ^18^ Furthermore, a report by Liao and colleagues revealed that the retraction of short‐term pressure overload rendered the heart resist pathological cardiac hypertrophy and heart failure progression, which was called myocardial hypertrophic preconditioning.^19^ Based on these studies, brief pressure overload may stimulate endogenous protective mechanisms and render the heart resistant to subsequent prolonged hypertrophic stimulation or ischaemic injury.^20^, ^21^ However, the potential mechanisms are still largely unknown. We hypothesize that the removal of transient pressure overload may induce resistance to subsequent myocardial ischaemic challenge and slow the progression to heart failure. In this study, we provided the first direct evidence that hypertrophic preconditioning conferred protective effects against I/R injury and involved improvements in mitochondrial autophagy.

## 2 METHODS

### 2.1 Animals

Male C57BL/6N mice were supplied by the Shanghai Laboratory Animal Center. The Sirt3‐knockout (SIRT3KO) mice were a kind gift from Dr Shen Weili of Shanghai Jiao Tong University (Shanghai, China). All protocols used conformed to the Guide for the Care and Use of Laboratory Animals published by the US National Institutes of Health (8th Edition, NRC 2011) and were approved by the Institutional Review Board of Zhongshan Hospital at Fudan University.

### 2.2 Grouping and treatment

The cardioprotective effects of hypertrophic preconditioning against I/R were assessed in four groups: (1) Sham group mice underwent a sham operation; (2) I/R group mice underwent I/R; (3) T3D4 group mice underwent aortic debanding after 3 days of transverse aortic constriction (TAC) without I/R; and (4) T3D4+I/R group mice underwent aortic debanding after 3 days of TAC, followed by I/R.

The effects of SIRT3 deficiency on hypertrophic preconditioning‐induced cardioprotection were assessed in eight groups: (1) Sham group mice underwent sham operation; (2) I/R group mice underwent I/R; (3) T3D4 group mice underwent aortic debanding after 3 days of TAC without I/R; (4) T3D4+I/R group mice underwent aortic debanding after 3 days of TAC, followed by I/R; (5) in the SIRT3KO+Sham group, SIRT3KO mice underwent a sham operation; (6) in the SIRT3KO+I/R group, SIRT3‐KO mice underwent I/R; (7) in the SIRT3KO+T3D4 group, SIRT3‐KO mice underwent aortic debanding after 3 days of TAC; and (8) in the SIRT3KO+T3D4+I/R group, SIRT3‐KO mice underwent aortic debanding after 3 days of TAC, followed by I/R.

To determine whether direct SIRT3 activation protected the heart from I/R injury, SIRT3 adenovirus (Adv‐SIRT3, 4 × 10^9^ IFU/mL) or control virus (Adv‐EGFP, 4 × 10^9^ IFU/mL) was injected into the left ventricular free wall using a 30‐gauge needle (three sites, 10 µL/site) in the following four groups: (1) Sham group mice underwent a sham operation; (2) I/R group mice underwent I/R; (3) Adv‐EGFP group mice were given control virus (Adv‐EGFP, intramyocardial injection 4 days before I/R) and underwent I/R; and (4) Adv‐SIRT3 group mice were given the SIRT3 adenovirus (Adv‐SIRT3, intramyocardial injection 4 days before I/R) and underwent I/R.

To examine the effects of mROS elimination on hypertrophic preconditioning‐induced cardioprotection, eight groups were used: (1) in the Sham+Vehicle group, sham‐operated mice were treated with 0.5% DMSO; (2) in the I/R+Vehicle group, ischaemic‐reperfused mice were treated with 0.5% DMSO; (3) in the T3D4+Vehicle group, aortic debanding was performed after 3 days of TAC in mice treated with 0.5% DMSO; (4) in the T3D4+I/R+Vehicle group, aortic debanding was performed after 3 days of TAC in mice treated with 0.5% DMSO before I/R; (5) in the Sham+MnTBAP group, sham‐operated mice were treated with MnTBAP (10 mg/kg, intraperitoneally 30 minutes before I/R); (6) in the I/R+MnTBAP group, ischaemic‐reperfused mice were treated with MnTBAP; (7) in the T3D4+ MnTBAP group, aortic debanding was performed after 3 days of TAC in mice treated with MnTBAP; and (8) in the T3D4+I/R+MnTBAP group, aortic debanding was performed after 3 days of TAC in mice treated with MnTBAP before I/R.

To examine the role of Beclin 1‐dependent autophagy in hypertrophic preconditioning‐induced cardioprotection, twelve groups were used: (1) Sham+Lenti‐EGFP group mice were administered only the control virus (lenti‐EGFP, intramyocardial injection 4 days before operation); (2) I/R+Lenti‐EGFP group mice were administered lenti‐EGFP 4 days before I/R; (3) in the T3D4+Lenti‐EGFP group, aortic debanding was performed after 3 days of TAC in mice treated with lenti‐EGFP; (4) in the T3D4+I/R+Lenti‐EGFP group, aortic debanding was performed after 3 days of TAC in mice treated with lenti‐EGFP before I/R; (5) Sham+Lenti‐shBeclin 1 group mice were administered only lentiviral Beclin 1 shRNA (lenti‐shBeclin 1, intramyocardial injection 4 days before operation); (6) I/R+Lenti‐shBeclin 1 group mice were administered lenti‐shBeclin 1 before I/R; (7) in the T3D4+Lenti‐shBeclin 1 group, aortic debanding was performed after 3 days of TAC in mice treated with lenti‐shBeclin 1; (8) in the T3D4+I/R+Lenti‐shBeclin 1 group, aortic debanding was performed after 3 days of TAC in mice treated with lenti‐shBeclin 1 before I/R; (9) Sham+Lenti‐Beclin 1 group mice were administered only the Beclin 1 lentivirus (lenti‐Beclin 1, intramyocardial injection 4 days before operation); (10) I/R+Lenti‐Beclin 1 group mice were administered lenti‐Beclin 1 before I/R; (11) in the T3D4+Lenti‐Beclin 1 group, aortic debanding was performed after 3 days of TAC in mice treated with lenti‐Beclin 1; and (12) in the T3D4+I/R+Lenti‐Beclin 1 group, aortic debanding was performed after 3 days of TAC in mice treated with lenti‐Beclin 1 before I/R.

To determine the effects of hypertrophic preconditioning on myocardial autophagic flux during I/R, eight groups were used: (1) in the Sham+Vehicle group, sham‐operated mice were treated with 0.5% DMSO; (2) in the I/R+Vehicle group, ischaemic‐reperfused mice were treated with 0.5% DMSO; (3) in the T3D4+Vehicle group, aortic debanding was performed after 3 days of TAC in mice treated with 0.5% DMSO; (4) in the T3D4+I/R+Vehicle group, aortic debanding was performed after 3 days of TAC in mice treated with 0.5% DMSO before I/R; (5) in the Sham+CQ group, sham‐operated mice were treated with chloroquine (CQ, 10 mg/kg, intraperitoneally 60 minutes before I/R); (6) in the I/R+CQ group, ischaemic‐reperfused mice were treated with CQ; (7) in the T3D4+CQ group, aortic debanding was performed after 3 days of TAC in mice treated with CQ; and (8) in the T3D4+IR+CQ group, aortic debanding was performed after 3 days of TAC in mice treated with CQ before I/R. MnTBAP, a stable and cell‐permeable superoxide dismutase mimetic and peroxynitrite decomposition catalyst, (10 mg/kg; Cayman Chemical) was administered intraperitoneally (ip) 30 minutes before coronary artery occlusion.^22^, ^23^ Calcitriol, an autophagosome‐lysosome fusion inhibitor, (CQ, 10 mg/kg; Cayman Chemical) was administered intraperitoneally (ip) 60 minutes before coronary artery occlusion.^24^ A 30‐µL aliquot of SIRT3 adenovirus (Adv‐SIRT3, 4 × 10^9^ IFU/mL) or the control virus (Adv‐EGFP, 4 × 10^9^ IFU/mL) was injected into the left ventricular free wall using a 30‐gauge needle (three sites, 10 µL/site). Beclin 1 lentivirus (lenti‐Beclin 1, 30 µL, 4 × 10^9^ IFU/mL), lentiviral Beclin 1 shRNA (lenti‐shBeclin 1, 30 µL, 4 × 10^9^ IFU/mL) or the control virus (lenti‐EGFP, 30 µL, 4 × 10^9^ IFU/mL) was injected into the left ventricular free wall using a 30‐gauge needle (three sites, 10 µL/site). The expression of SIRT3 and Beclin 1 in the myocardium was determined at 4 days post‐injection. The surgeon was blinded to the treatment allocations.

### 2.3 Transverse aortic constriction‐induced hypertrophic preconditioning

Male C57BL/6 mice (8 weeks, 22‐25 g) were subjected to minimally invasive TAC, debanding or sham operation as described elsewhere.^25^ The mice were anesthetized with a single intraperitoneal injection of 1% pentobarbital sodium (50 mg/kg). A horizontal skin incision approximately 1 cm in length was made at the level of the suprasternal notch. Once the trachea was located, a 5‐mm longitudinal cut was made down the sternum, the thymus was retracted, and the aortic arch was located with the help of a retractor. A wire with a snare on the end was passed under the aorta between the origin of the right innominate and left common carotid arteries. A 6‐0 silk suture was snared with the wire and pulled back around the aorta. A bent 27‐gauge needle was then placed next to the aortic arch, and the suture was snugly tied around the needle and the aorta. After ligation, the needle was quickly removed. The skin was closed, and mice were allowed to recover on a warming pad until they were fully awake. The sham procedure was identical except that the aorta was not ligated. The debanding operation was performed by carefully removing the ligature after 3 days of TAC, and then, myocardial infarction surgery was performed 4 days later. After the mice recovered from general anaesthesia, the animals were subcutaneously injected with meloxicam (5 mg/kg) to mitigate pain.

### 2.4 Myocardial ischaemia‐reperfusion protocol

The surgical procedures were performed as previously described.^26^ Briefly, mice were anesthetized with 2% isoflurane, and the heart was manually exposed without intubation via a tiny thoracic incision. A ligation was made around the left anterior descending coronary artery 2‐3 mm from its origin using a 6‐0 silk suture, and then, the chest was closed. After 40 minutes of coronary artery occlusion, the suture was released followed by reperfusion for 24 hours. Mice that fully recovered from the surgical procedure were returned to standard animal housing conditions.

### 2.5 In vivo adenoviral‐mediated cardiac‐specific gene overexpression

The adenoviral shuttle vector GV135‐CMV‐EGFP was obtained from Biowit Technologies, and the adenoviral backbone plasmids pBHGloxdeltaE1 and 3Cre were obtained from Microbix Biosystems, Inc Murine SIRT3 cDNA was cloned into the adenoviral shuttle vector to generate GV135‐CMV‐SIRT3‐EGFP vectors. The SIRT3 adenoviruses were generated according to the instructions of the AdMax™ Adenoviral Vector Creation System, and the resulting viral titres were determined using the Adeno‐X‐Rapid Titer Kit (BD Biosciences Clontech). The control virus (Adv‐EGFP) carried the coding sequence for expressing green fluorescent protein (EGFP) with an empty coding sequence for SIRT3. The mice were anesthetized with 2% isoflurane, and the heart was exposed via a left thoracotomy at the fifth intercostal space. Adenovirus (4 × 10^9^ IFU/mL) was administered by a direct injection in the left ventricular free wall (three sites, 10 µL/site, 32.5‐gauge needle). Myocardial target gene expression was analysed 4 days after virus injection as previously described.^24^

### 2.6 In vivo lentiviral‐mediated cardiac‐specific gene delivery

The lentiviral plasmids encoding shRNAs for Beclin 1 (forward: 5′‐CcggGCTGGACACTCAGCTCAATTTCAAGAGAATTGAGCTGAGTGTCCAGCTTTTTTg‐3′; reverse: 5′‐ aattcaaaaaaGCTGGACACTCAGCTCAATTCTCTTGAAATTGAGCTGAGTGTCCAGC‐3′) were constructed in the pLKD‐CMV‐eGFP‐U6‐shRNA vector, which was purchased from Obio Technologies, Inc. A plasmid carrying a non‐targeting sequence was used to create the control cells. For virus packaging, the control or Beclin 1‐specific shRNA constructs were cotransfected with Mission lentiviral packing mix. The recombinant Beclin‐1 overexpression plasmid expressing EGFP was constructed using the pLenti‐CMV‐EGFP‐P2A‐MCS‐3FLAG vector and packaged into the lentivirus by Obio Technologies, Inc. The titres of the stocks were measured by plaque assays and were 2.13 × 10^9^ IU/mL for lenti‐Beclin 1, 2.04 × 10^9^ IU/mL for lenti‐shBeclin 1 and 2.21 × 10^9^ IU/mL for lenti‐EGFP. The mice were anesthetized with 2% isoflurane, and the heart was exposed via a left thoracotomy at the fifth intercostal space. Lenti‐Beclin, lenti‐shBeclin 1 or control virus was administered by direct injection in the left ventricular free wall (three sites, 10 µL/site, 32.5‐gauge needle). Myocardial target gene expression was analysed 4 days after virus injection.^27^

### 2.7 Doppler echocardiography

The mouse was anesthetized with 1% isoflurane after 24 hours of reperfusion. M‐mode images of the left ventricular long axis were obtained at the level of the papillary muscle tips using a Vevo 770 imaging system (VisualSonics). The left ventricular internal diastolic diameter (LVIDd) and left ventricular internal systolic diameter (LVIDs) were recorded. Left ventricular fractional shortening (LVFS) was calculated according to the following formula: $\text{LVFS}=\left[\left(\text{LVIDd}‐\text{LVIDs}\right)/\text{LVIDd}\right]\times 100$. Left ventricular ejection fraction (LVEF) was calculated by using the spherical formula.

### 2.8 Determination of infarct size

Myocardial infarct size was assessed by 2,3,5‐triphenyltetrazolium chloride (TTC, Sigma‐Aldrich) and Evans blue staining after reperfusion for 24 hours. Briefly, the coronary artery was religated, and 0.2 mL of 2% Evans blue dye was injected into the right ventricular cavity to identify the unstained area as the area at risk. The hearts were harvested and frozen, sectioned into 2‐mm slices and stained in 1% TTC solution at 37°C for 10 minutes to identify the infarct zone (pale) and area at risk (red). The myocardial infarction size (IS) is expressed as a percentage of the area at risk was analysed using ImageJ 1.37 software (National Institutes of Health).

### 2.9 Detection of myocardial apoptosis

Myocardial apoptosis was assessed by terminal deoxynucleotidyl transferase dUTP nick‐end labelling (TUNEL) staining using a fluorescein in situ cell death detection kit (Roche) as described elsewhere. Green fluorescein staining indicates apoptotic nuclei. TUNEL‐positive nuclei (green nuclei) are expressed as the percentage of the total cell population.

### 2.10 Detection of Caspase‐3 activity in heart tissue

Myocardial Caspase‐3 activity was used to determine apoptosis levels using a Caspase fluorometric assay kit (BioVision) at 3 hours after reperfusion. Briefly, 100 μg of total tissue protein per assay and a final concentration of 50 μmol/L caspase‐3‐specific AFC‐conjugated substrates (IEDT, LEHD and ATAD) were loaded. The samples were measured by a fluorimeter equipped with a 400‐nm excitation and a 505‐nm emission filter. Caspase‐3 activity was calculated against the mean value of Caspase‐3 activity from the corresponding control.

### 2.11 Measurement of superoxide production

Superoxide production was assessed by DHE and MitoSOX staining. Five‐micrometre‐thick frozen slices without fixation were stained with DHE or MitoSOX at 37℃ for 30 minutes. Photographs were acquired using a fluorescence microscope. Fluorescence intensity was assessed by using ImageJ 1.37.

### 2.12 Mitochondrial isolation

Myocardial mitochondria were isolated by using a mitochondria isolation kit (Beyotime Biotechnology) according to the manufacturer's protocol. Briefly, the samples from the areas at risk were harvested at 3 hours after reperfusion and were cut into small pieces followed by incubation in trypsin for 20 minutes at 4°C. Then, the trypsin was removed and the mitochondrial extraction buffer was added. After grinding for 30 times, the homogenates were centrifuged at 600 *g* for 5 minutes to harvest the supernatant. Finally, the supernatant was centrifuged at 11 000 *g* for 10 minutes to collect the mitochondria.

### 2.13 Mitochondrial SIRT3 and SOD2 activity assessment

Myocardial mitochondria were isolated at 3 hours after reperfusion. SIRT3 activity was determined by using a SIRT3 fluorescent assay kit (BPS Bioscience), and superoxide dismutase 2 (SOD2) activity was quantified by using a mitochondrial SOD2 activity assay kit (Beyotime Biotechnology) according to the manufacturer's protocol.

### 2.14 Autophagosome morphology assessment

After reperfusion for 3 hours, the samples harvested from the areas at risk were fixed with 2% glutaraldehyde for 2 hours, fixed in 1% OsO4 for 2 hours and embedded in resin. The ultrathin sections were stained with uranyl acetate and lead citrate and observed under an electron microscope (Philips CM‐120; Philips Electronic Instruments). Random images were acquired by electron microscopy.

### 2.15 Immunoblotting

Samples were collected from the area at risk after reperfusion for 3 hours. The expression of myocardial LC3B (1:1000, Cell Signaling Technology), SIRT3 (1:1000, CST, USA), acetylated SOD2 (lysine 68, 1:1000, Abcam, USA), SOD2 (1:1000, Santa Cruz, USA), COX4 (1:1000, CST, USA), p62 (1:1000, CST, USA), Beclin 1 (1:1000, CST, USA) and GAPDH (1:1000, CST, USA) was determined by immunoblotting.^28^ The quantitative protein band density was measured by ImageJ 1.37.

### 2.16 Statistical analysis

The data are shown as the means ± SD. Statistical analysis was performed with unpaired Student's *t* tests for two‐group comparisons. For multigroup comparisons, one‐way ANOVA followed by the Bonferroni post hoc test was used. A value of *P* < .05 was considered to be statistically significant. All statistical analyses were performed using GraphPad Prism Version 7.0 (GraphPad Prism Software).

## 3 RESULTS

### 3.1 Hypertrophic preconditioning inhibited myocardial I/R‐induced cell death and cardiac dysfunction

**FIGURE 1:**
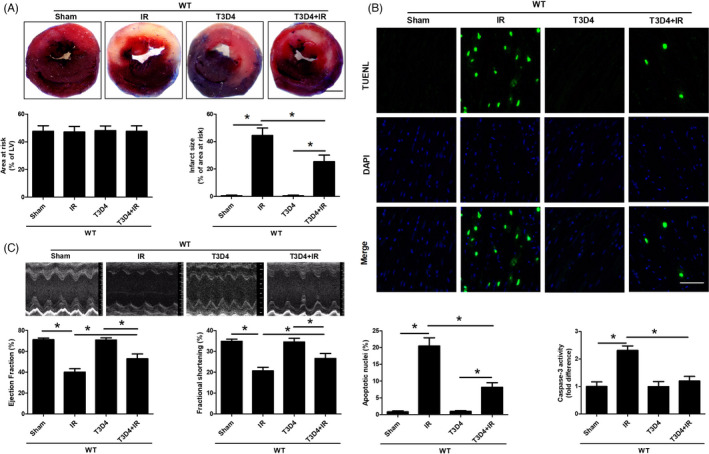
Hypertrophic preconditioning decreased myocardial infarct size, inhibited cardiomyocyte apoptosis and improved cardiac function. A, Myocardial infarct size, as determined by Evans blue and TTC staining at 24 h after I/R; Scale bar = 1 mm. B, Cardiomyocyte apoptosis, as assessed by TUNEL staining (400×) and caspase‐3 activity measurement following in situ I/R; scale bar = 50 μm. C, Cardiac function (LVEF and LVFS), as measured by Doppler echocardiography at 24 h after I/R. n = 6‐8 per group, *P < .05

Transient TAC was performed for 3 days in male mice and then withdrawn for 4 days, followed by subsequent exposure to regional myocardial ischaemia by coronary artery ligation to determine whether the retraction of transient hypertrophic stimulation rendered the heart resistant to subsequent ischaemic injury. Preconditioning with TAC for 3 days induced mild cardiac hypertrophy, as shown by an increased heart weight/body weight ratio and heart weight/tibial length ratio (Figure S1), which may be considered compensatory hypertrophy. TAC for 3 days markedly reduced I/R‐induced necrotic cardiomyocyte death as assessed by infarct size (25.38% ± 4.73% in the T3D4+I/R group vs 44.43% ± 5.41% in the I/R group, *P* < .05, Figure 1A), whereas the area at risk did not show marked differences among the groups. Moreover, transient aortic banding inhibited I/R‐induced myocardial apoptotic responses, as evidenced by a reduction in the number of TUNEL‐positive nuclei and caspase‐3 activity (Figure 1B). Cardiac function was measured by echocardiography at 24 hours after reperfusion to assess LV performance. We found that I/R markedly reduced the LVEF and LVFS, whereas the removal of short‐term aortic banding alleviated the I/R‐induced declines in LVEF and LVFS (Figure 1C). Taken together, these results demonstrated that hypertrophic preconditioning inhibited cardiac I/R‐induced cell death and cardiac dysfunction.

### 3.2 Hypertrophic preconditioning activated SIRT3 and suppressed I/R‐induced autophagy

**FIGURE 2:**
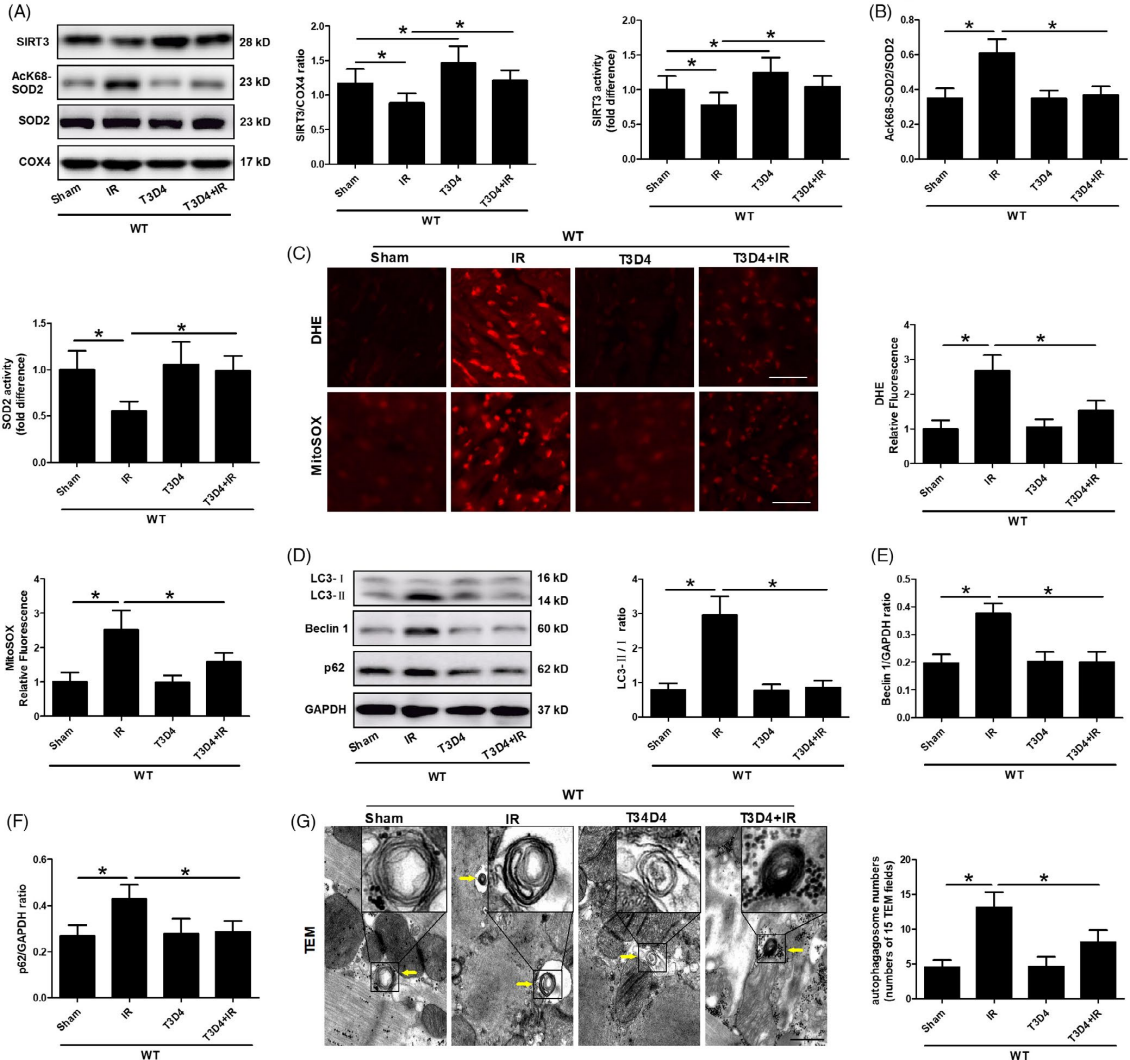
Hypertrophic preconditioning suppressed I/R‐induced autophagy. A, Myocardial SIRT3 protein expression and activity. B, Myocardial acetylated SOD2 levels and activity. COX4 was used as a loading control for mitochondrial proteins. C, Quantitative analysis of DHE and MitoSOX fluorescence intensity; scale bar = 50 μm. D, The expression of LC3B in I/R‐induced myocardial tissue, as detected by immunoblotting. E, Beclin 1. F, p62. GAPDH was used as a loading control. G, Representative autophagosome images indicated by the yellow arrows, and the number of autophagosomes was quantified by analysing 15 fields in each sample; scale bar = 500 nm. n = 5‐6 per group, *P < .05

SIRT3, a major mitochondrial deacetylase, plays a critical role in regulating mitochondrial autophagy by deacetylating SOD2.^29^ The role of SIRT3 in regulating protein deacetylation and its involvement in diverse physiological and pathophysiological conditions has suggested that this deacetylase is a potential therapeutic target. However, the roles of SIRT3 activation and protein deacetylation in I/R injury, particularly in the context of hypertrophic preconditioning, remain unclear. Both the expression and activity of SIRT3 were significantly decreased in the I/R group after myocardial I/R injury but were significantly increased in the T3D4+I/R group at 24 hours after I/R injury (Figure 2A). We analysed the acetylation status of the SIRT3 substrate SOD2 using an antibody that specifically detects SOD2 acetylation at K68 to assess whether increased SIRT3 levels were associated with increased SIRT3 activity. SOD2 activity has been shown to be increased by SIRT3‐mediated deacetylation at K68.^29^ Consistent with the increased SIRT3 levels, we observed increased SIRT3 activity, as revealed by reduced SOD2 acetylation following the removal of short‐term pressure overload in mice with I/R injury (Figure 2B). SOD2 activity is tightly regulated by the acetylation of its lysine residues. Therefore, we assessed myocardial SOD2 activity. Compared with the sham‐operated group, the I/R group showed a marked reduction in SOD2 activity, whereas transient TAC promoted SOD2 activation and deacetylation (Figure 2B). Moreover, we observed markedly reduced mitochondrial‐derived superoxide production as assessed by DHE and MitoSOX staining (Figure 2C). Next, we investigated the role of mROS‐mediated autophagy in hypertrophic preconditioning and assessed LC3 conversion from the soluble form (LC3‐I) to the cleaved autophagosome‐associated form (LC3‐II) and Beclin 1 activation during I/R. We found that the ratio of LC3‐II/LC3‐I and Beclin 1 activation were markedly enhanced by I/R (Figure 2D,E), suggesting exacerbated autophagy and augmented autophagosome accumulation. Notably, I/R did not significantly reduce the protein expression p62 (a specific autophagic substrate protein that represents autophagic flux) (Figure 2F). Compared with those in non‐preconditioned mice, hypertrophic preconditioning markedly reduced the conversion of LC3‐I to LC3‐II (Figure 2D), inhibited Beclin 1 activation (Figure 2E), downregulated p62 expression (Figure 2F) and reduced autophagosome accumulation (Figure 2G). These findings demonstrated that myocardial hypertrophic preconditioning was sufficient to activate SIRT3‐SOD2 signalling and hinder I/R‐induced autophagy.

### 3.3 SIRT3 deficiency reversed the cardioprotective effects of hypertrophic preconditioning

**FIGURE 3:**
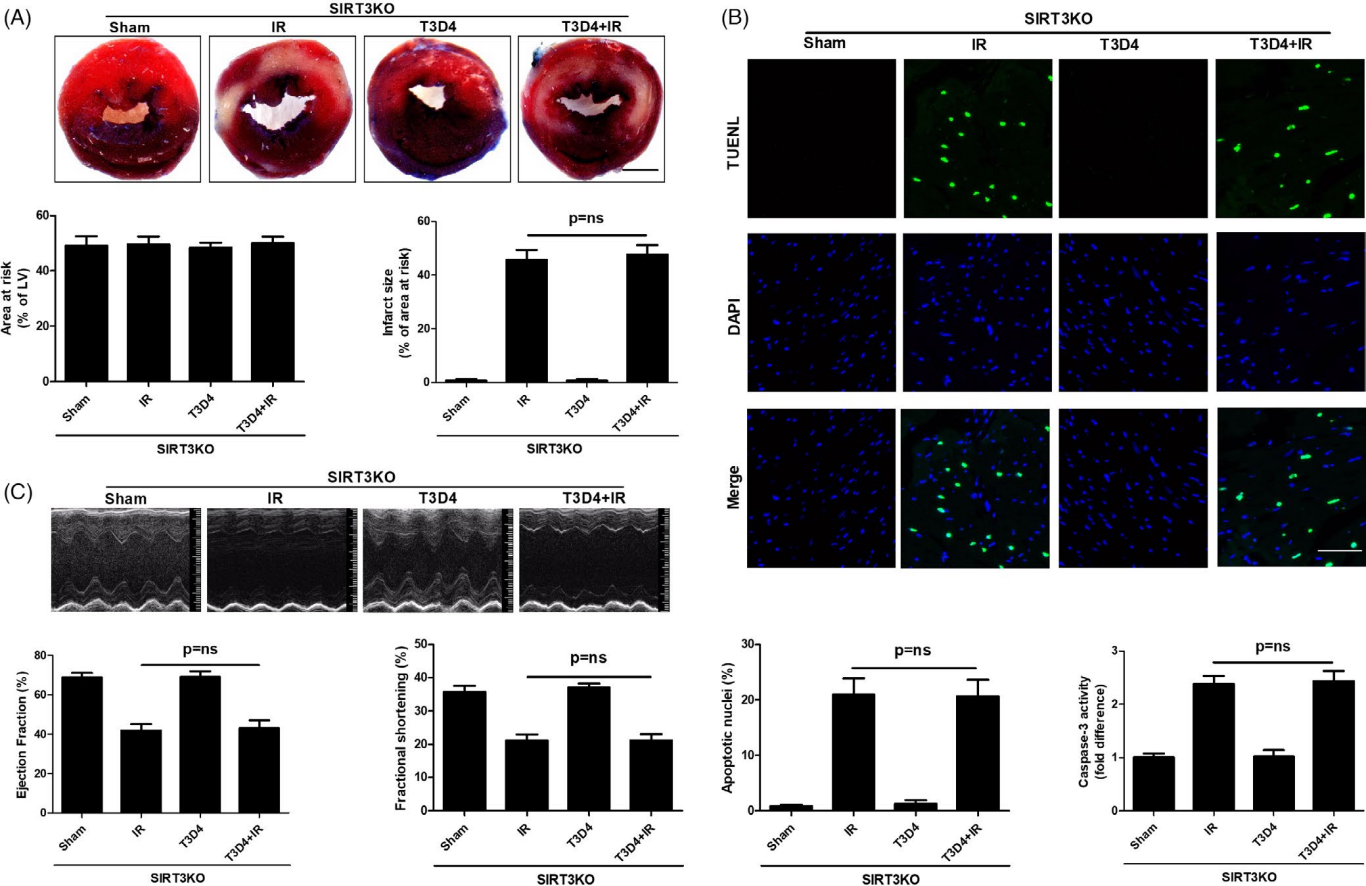
SIRT3 deficiency reversed the cardioprotective effects of hypertrophic preconditioning. A, Myocardial infarct size, as determined by Evans blue and TTC staining at 24 h after I/R; Scale bar = 1 mm. B, Cardiomyocyte apoptosis, as assessed by TUNEL staining (400×) and caspase‐3 activity analysis following in situ I/R; scale bar = 50 μm. C, Cardiac function (LVEF and LVFS), as measured by Doppler echocardiography at 24 h after I/R. n = 6‐8 per group, *P < .05

We examined myocardial morphology and function in response to I/R injury in the presence or absence of hypertrophic preconditioning in SIRT3‐KO mice to demonstrate that hypertrophic preconditioning was involved in I/R‐induced autophagy and the disruption of SIRT3‐mediated mitochondrial‐derived superoxide generation. Myocardial SIRT3 deficiency was verified by immunoblotting (Figure S2). Myocardial infarct size (Figures 1A,3A), cardiomyocyte apoptosis (Figures 1B,3B) and cardiac function (Figures 1C,3C) were not significantly different between WT and SIRT3‐KO mice following I/R. Interestingly, genetic ablation of SIRT3 markedly eliminated the hypertrophic preconditioning effects, as evidenced by expanded myocardial infarct size (Figure 3A), exacerbated cardiomyocyte apoptosis (Figure 3B) and worsened cardiac function in I/R mouse hearts (Figure 3C). Based on these findings, SIRT3 is required for hypertrophic preconditioning‐induced cardioprotection against I/R injury.

### 3.4 SIRT3 was required for hypertrophic preconditioning‐mediated regulation of the mROS‐autophagy pathway

**FIGURE 4:**
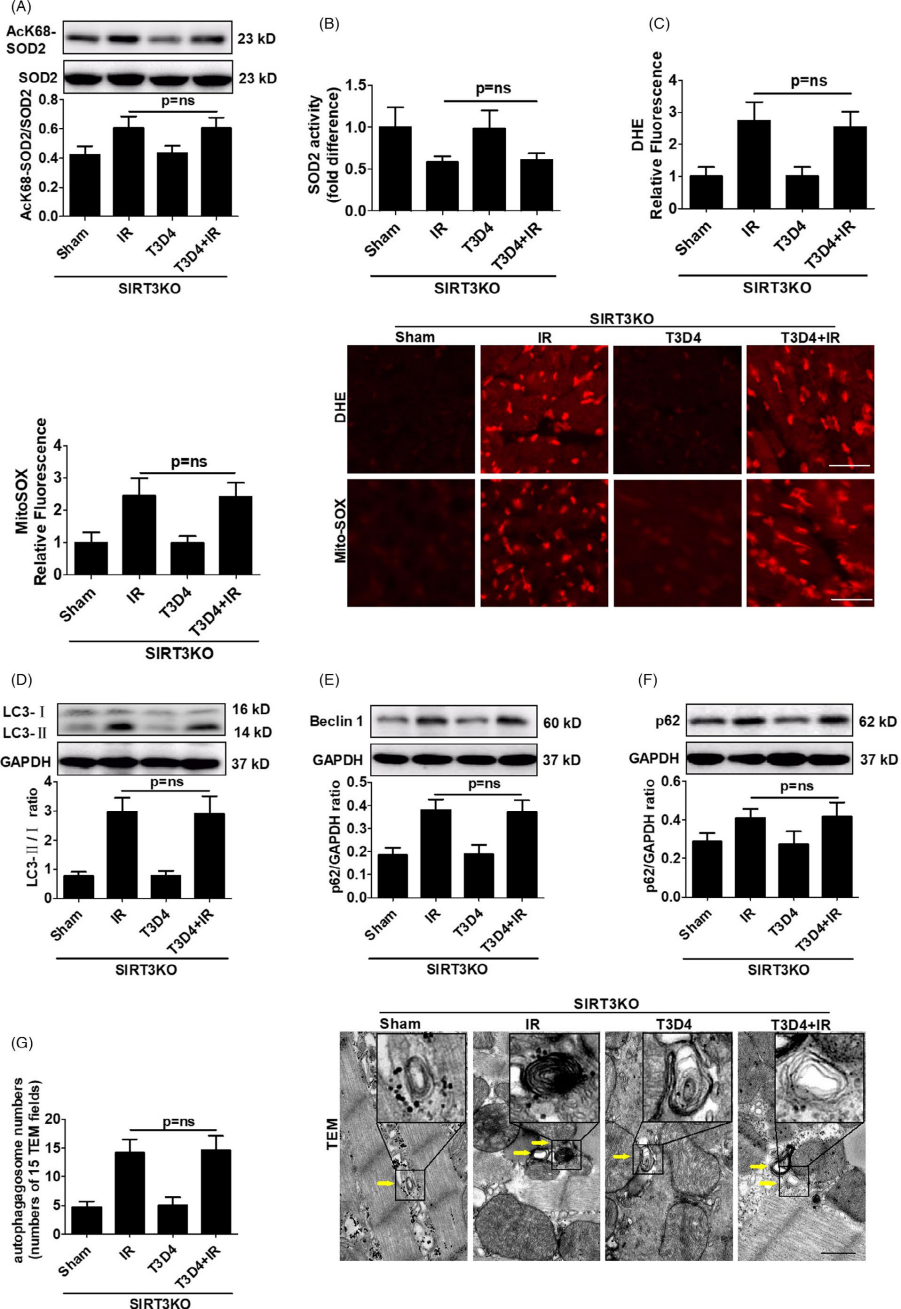
Endogenous SIRT3 was required for the regulatory effects of hypertrophic preconditioning on the mROS‐autophagy pathway. A, The level of acetylated SOD2 in the I/R‐induced myocardium. B, SOD2 activity. C, Quantitative analysis of DHE and MitoSOX fluorescence intensity; scale bar = 50 μm. D, The expression of LC3B in I/R‐induced myocardial tissue, as detected by immunoblotting. E, Beclin 1. F, p62. G, Representative autophagosome photographs indicated by the yellow arrows, and the number of autophagosomes was quantified by analysing 15 fields in each sample; scale bar = 500 nm. n = 5‐6 per group, *P < .05

SIRT3‐deficient mice were used to determine whether SIRT3 is involved in hypertrophic preconditioning‐mediated regulation of the mROS‐autophagy pathway. We hypothesized that SIRT3 deficiency could abrogate the preconditioning‐induced improvement in myocyte autophagy. Indeed, the hypertrophic preconditioning‐induced decreases in SOD2 acetylation levels (Figure 4A) and increases in SOD2 activity (Figure 4B) were significantly attenuated by genetic ablation of SIRT3 in I/R‐induced mice. Moreover, we demonstrated that the hypertrophic preconditioning‐induced decreases in mitochondrial‐derived superoxide production and autophagic stress were reversed by SIRT3 ablation, as shown by increased DHE and MitoSOX fluorescence intensity (Figure 4C), which promoted the conversion of LC3‐I to LC3‐II (Figure 4D), upregulated Beclin 1 expression (Figure 4E), increased p62 activation (Figure 4F) and augmented autophagosome accumulation (Figure 4G) in the I/R‐induced myocardium. Myocardial LC3‐II/ LC3‐I ratio (Figures 2D,4D), Beclin 1 expression (Figures 2E,4E), p62 expression (Figures 2F,4F) and autophagosome numbers (Figures 2G,4G) in the I/R‐induced myocardium were not significantly different between WT and SIRT3‐KO mice with or without I/R. Taken together, these findings suggest that SIRT3 is required for hypertrophic preconditioning‐mediated regulation of the mROS‐autophagy pathway.

### 3.5 An adenovirus encoding SIRT3 mimicked the effects of hypertrophic preconditioning on myocardial I/R

**FIGURE 5:**
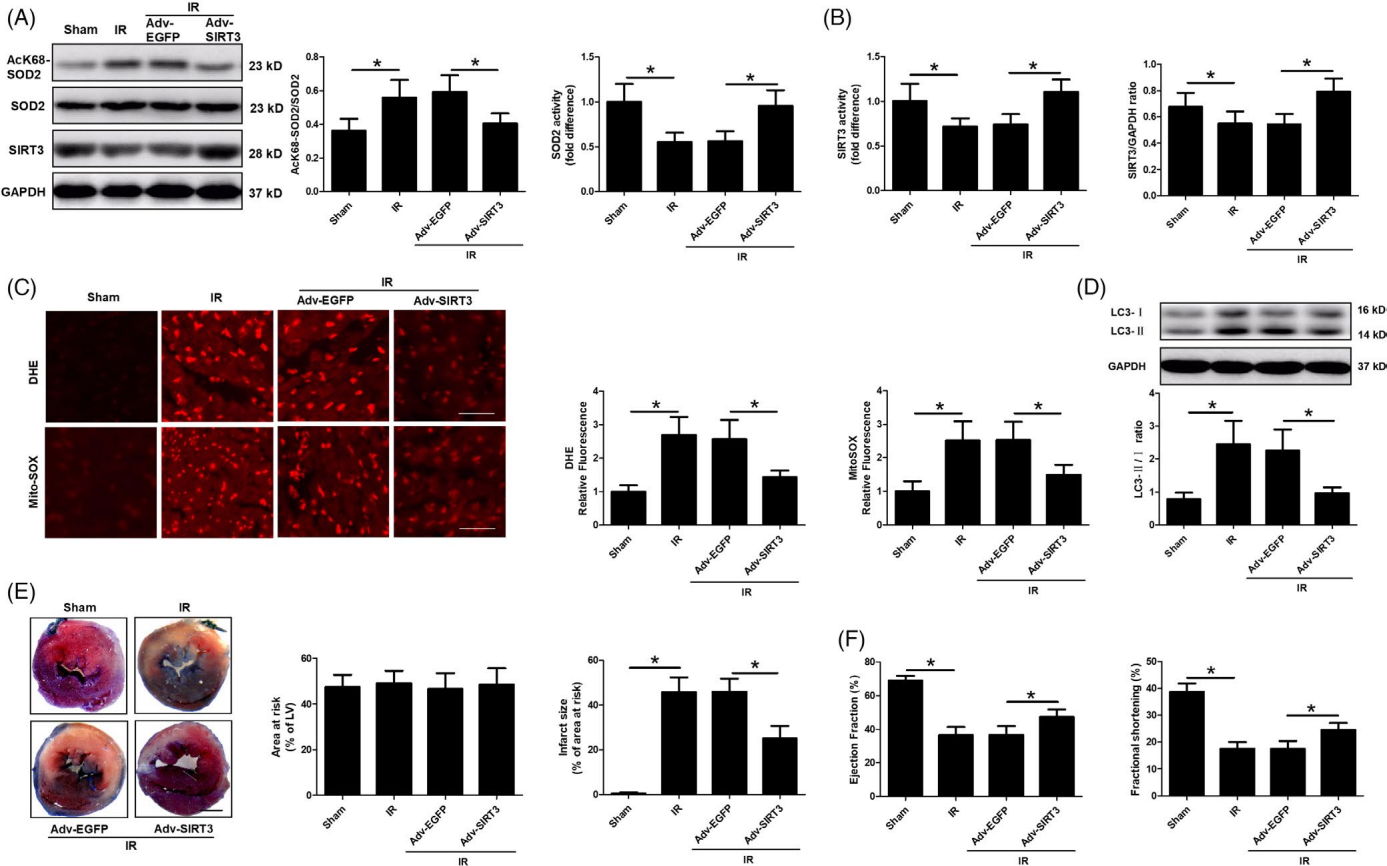
SIRT3 deacetylated SOD2 and modulated the mROS‐autophagy pathway. A, Myocardial SOD2 protein expression and activity. B, SIRT3 protein expression and activity. C, Quantitative analysis of DHE and MitoSOX fluorescence intensity; scale bar = 50 μm. D, Quantitative analysis of LC3B expression. E, Myocardial infarct size, as detected by Evans blue and TTC staining at 24 h after I/R; Scale bar = 1 mm. F, Cardiac function (LVEF and LVFS), as measured by Doppler echocardiography at 24 h after I/R. n = 6‐8 per group, *P < .05

The adenovirus encoding SIRT3 was administered to mice by direct intramyocardial injection four days before I/R to determine whether the restoration of SIRT3 expression was sufficient to hinder I/R‐induced autophagy and mimicked the effects of hypertrophic preconditioning. We found that SIRT3 overexpression decreased SOD2 acetylation and reduced SOD2 activity (Figure 5A). Moreover, SIRT3 overexpression attenuated the I/R‐induced reductions in SIRT3 protein expression and activity (Figure 5B). Furthermore, SIRT3 activation markedly suppressed mitochondrial‐derived superoxide production in mouse hearts exposed to I/R (Figure 5C). SIRT3 overexpression in the myocardium also induced a marked reduction in autophagy and resistance to ischaemic injury, as evidenced by a reduced LC3‐II/I ratio (Figure 5D), decreased myocardial infarct size (24.96% ± 5.78% in the Adv‐SIRT3 group *vs*. 46.04% ± 5.74% in the Adv‐EGFP group, *P* < .05, Figure 5E) and elevated LVEF and LVFS (Figure 5F). These results suggest that SIRT3 activation plays a pivotal role in the maintenance of cardiac survival and function by regulating the SOD2‐mROS pathway.

### 3.6 mROS scavenging ameliorated I/R injury and mediated the cardioprotective effects of hypertrophic preconditioning

**FIGURE 6:**
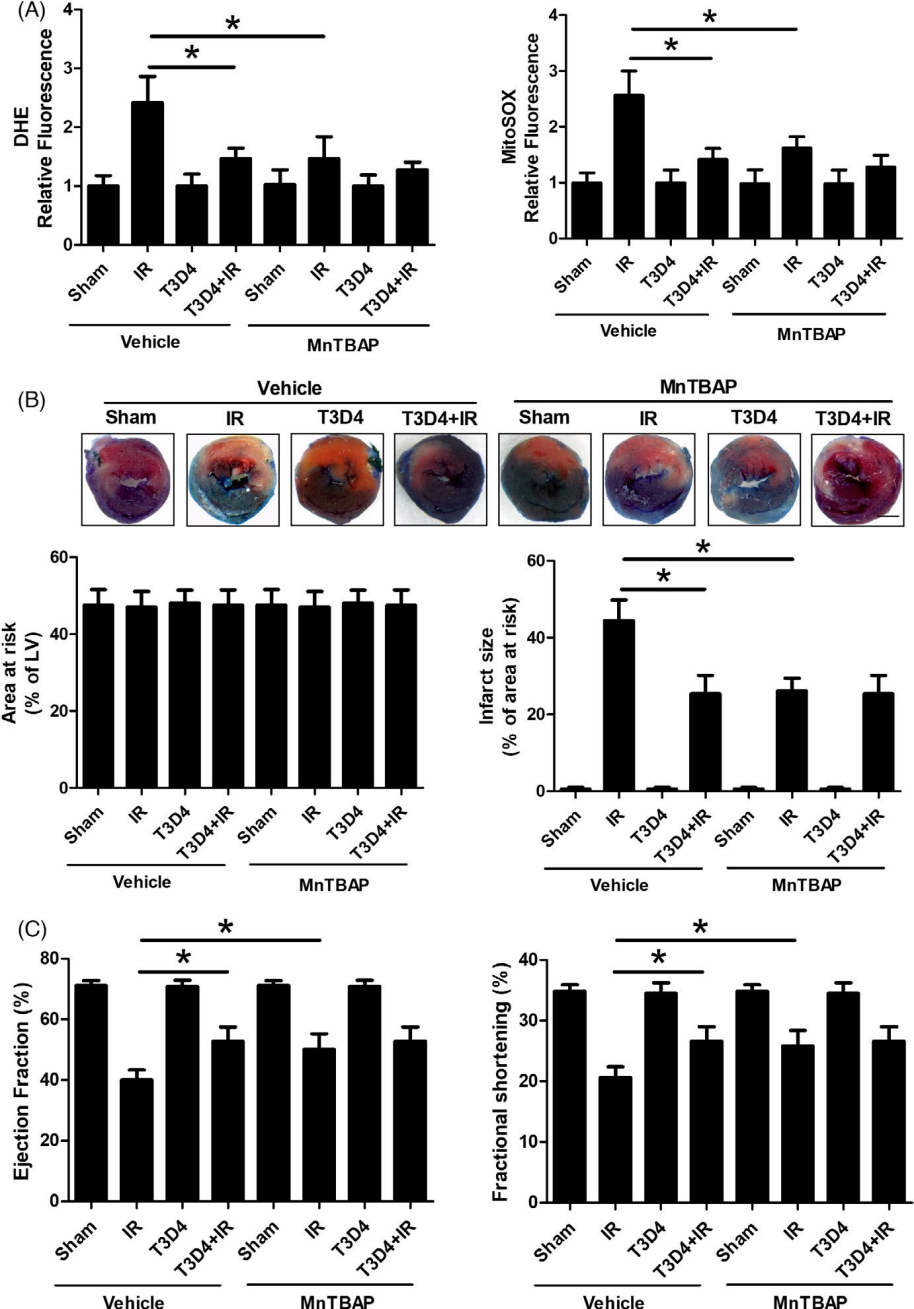
mROS scavenging rescued I/R injury and was involved in the cardioprotective effect of hypertrophic preconditioning. A, Quantitative analysis of DHE and MitoSOX fluorescence intensity. B, Myocardial infarct size, as determined by Evans blue and TTC staining at 24 h after I/R; Scale bar = 1 mm. C, Cardiac function (LVEF and LVFS), as measured by Doppler echocardiography at 24 h after I/R. n = 6‐8 per group, *P < .05

Next, we assessed the contribution of mitochondrial‐derived superoxide to the cardioprotective effect of hypertrophic preconditioning in I/R‐induced mouse hearts. MnTBAP was administered to the animal in the presence or absence of hypertrophic preconditioning. MnTBAP treatment mimicked SOD2 activity and prevented ROS production in I/R‐induced hearts (Figure 6A). Notably, the administration of MnTBAP to I/R‐induced mouse hearts exerted notable cardioprotective effects, as evidenced by diminished myocardial infarct size (Figure 6B) and improved cardiac function (Figure 6C). However, no additional cardioprotective effects were achieved when we combined MnTBAP and hypertrophic preconditioning in mice exposed to I/R **(**Figure 6B,C). These results indicate that the removal of short‐term pressure overload confers cardioprotection against I/R injury by inhibiting mitochondrial superoxide production.

### 3.7 The restoration of Beclin 1‐dependent autophagic flux contributed to the protective effects of hypertrophic preconditioning on myocardial infarction

**FIGURE 7:**
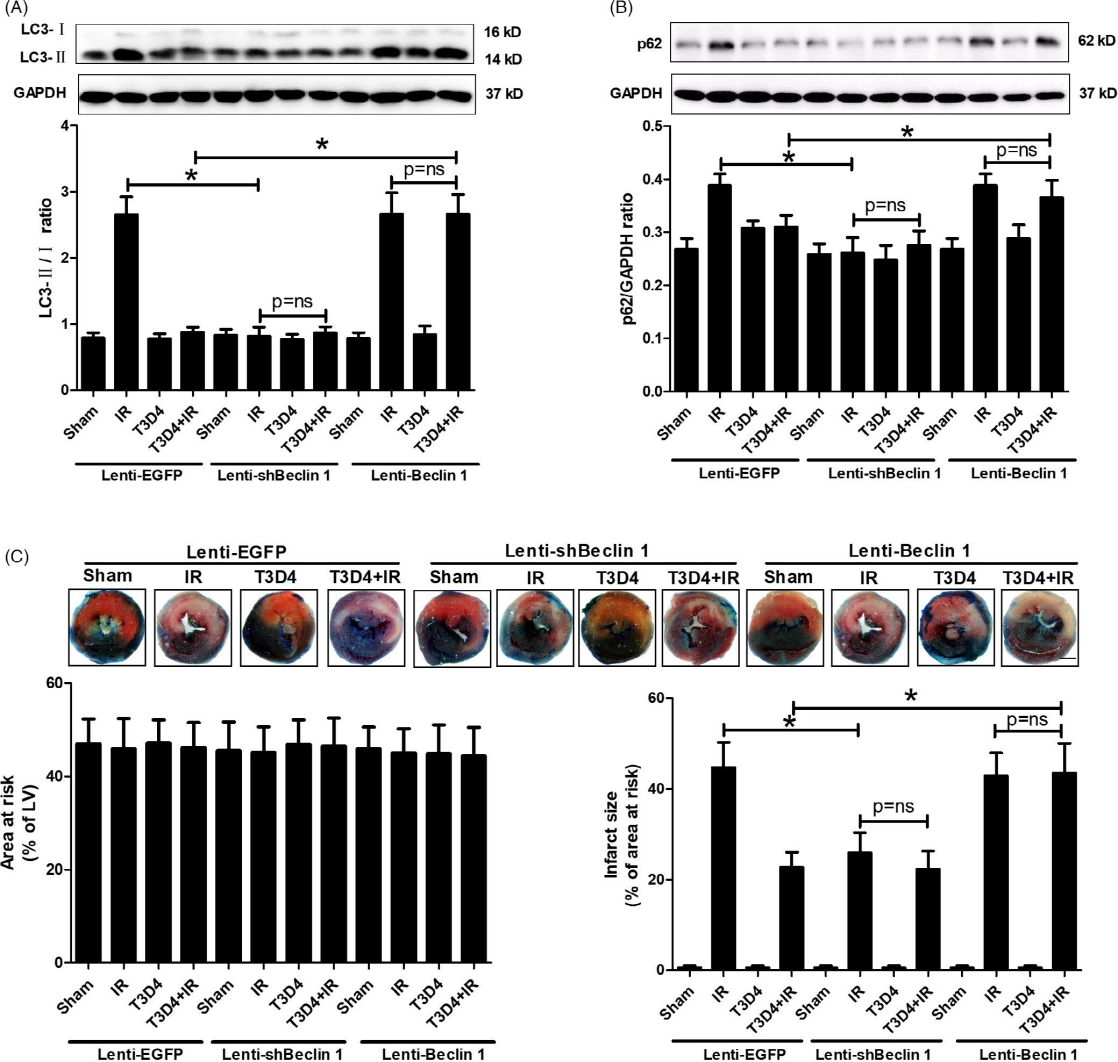
Beclin 1 eliminated the protective effect of hypertrophic preconditioning against myocardial infarction. A, The expression of LC3B in I/R‐induced myocardial tissue, as detected by immunoblotting. B, p62. C, Myocardial infarct size, as determined by Evans blue and TTC staining at 24 h after I/R; Scale bar = 1 mm. n = 6‐8 per group, *P < .05

**FIGURE 8:**
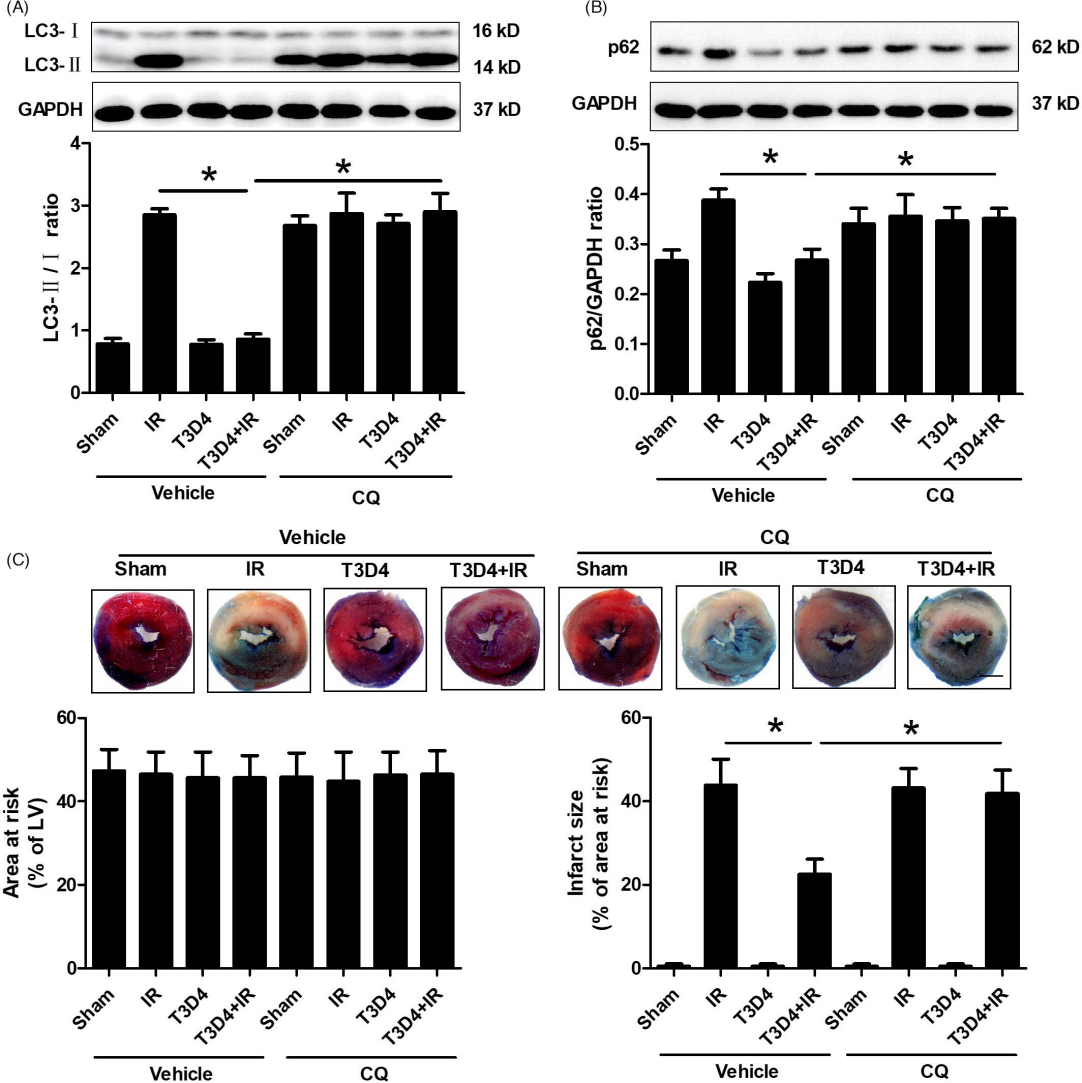
Chloroquine (CQ) attenuated hypertrophic preconditioning‐induced protection against myocardial infarction by regulating autophagic flux. A, Myocardial LC3B expression. B, p62. C, Myocardial infarct size; Scale bar = 1 mm. n = 6‐8 per group, *P < .05

Beclin 1, an autophagy‐associated tumour suppressor gene, has been shown to play a pivotal role in modulating autophagic cell death during I/R.^30^, ^31^ However, the role of Beclin 1 in hypertrophic preconditioning‐mediated protection against I/R is still unclear. To determine whether Beclin 1 inhibition contributes to hypertrophic preconditioning‐induced cardioprotection by regulating autophagosome‐lysosome fusion in the heart, lenti‐shBeclin 1, lenti‐Beclin 1 or control virus (lenti‐EGFP) was administered to mice by direct intramyocardial injection 4 days before aortic banding. Beclin 1 protein expression decreased nearly 40% in the lenti‐shBeclin 1‐infected myocardium and increased 43% in the lenti‐Beclin 1‐infected myocardium compared to the lenti‐EGFP‐infected myocardium at 4 days after intramyocardial injection of the lentivirus (Figure S3). Beclin 1 knockdown markedly inhibited I/R‐induced autophagic stress, as evidenced by diminished LC3B and p62 expression (Figure 7A,B), whereas Beclin 1 overexpression did not induce additional autophagic stress and reversed hypertrophic preconditioning‐induced attenuation of autophagy in the I/R‐stimulated myocardium (Figure 7A,B). Moreover, the protective effect of hypertrophic preconditioning against myocardial infarction was mimicked by Beclin 1 knockdown, as shown by the reduced myocardial infarct size in lenti‐shBeclin‐infected mouse hearts. However, Beclin 1 knockdown did not induce additional cardioprotective effects compared to hypertrophic preconditioning (Figure 7C). In contrast, Beclin 1 overexpression did not further promote I/R‐induced cardiomyocyte death but inhibited the protective effects of hypertrophic preconditioning against myocardial infarction in the I/R‐induced myocardium (Figure 7C). These findings demonstrated that Beclin 1‐dependent autophagic flux contributed to the protective effects of hypertrophic preconditioning against myocardial infarction. In addition, preventing autophagosome‐lysosome fusion using chloroquine (CQ) did not induce an additional increase in LC3‐II or p62 expression in non‐preconditioned mice (Figure 8A,B), indicating that I/R‐induced autophagic dysfunction and impaired autophagosome clearance. Compared with those in non‐preconditioned hearts, hypertrophic preconditioning markedly reduced the ratio of LC3‐II/LC3‐I and p62 expression, as well as the myocardial infarct size (Figure 8A,B). However, CQ pre‐treatment markedly increased the LC3‐II/LC3‐I ratio (Figure 8A), inhibited p62 activation (Figure 8B) and enhanced autophagic cell death (Figure 8C) in preconditioned mice, suggesting that autophagic flux was preserved by hypertrophic preconditioning. Taken together, these findings demonstrated a Beclin 1‐dependent mechanism by which hypertrophic preconditioning affects autophagic cell death in the I/R‐induced myocardium.

## 4 DISCUSSION

In this study, we presented new insights into the role of pressure overload‐induced preconditioning in the heart. The novel contributions of the present work can be summarized as follows. First, we reported that declines in SIRT3 expression and deacetylase activity inhibited SOD2 deacetylation, resulting in reduced SOD2 activity and elevated mROS, which contributed to I/R‐induced autophagy. Second, we demonstrated that the expression and deacetylase activity of SIRT3 contribute to SOD2 activity and the cardioprotective effects of hypertrophic preconditioning. Third, we provided the first direct evidence that hypertrophic preconditioning mitigated I/R‐induced autophagic cell death by modulating the SIRT3‐SOD2‐mROS pathway, thus representing a potential strategy for the treatment of ischaemic heart diseases.

Lysine acetylation is a critical post‐translational modification involved in the regulation of mitochondrial protein activity.^32^ Recently, SIRT3 has been identified as an important mitochondrial deacetylation enzyme that directly binds and deacetylates SOD2 (at lysine 68), thereby enhancing SOD2 activity and contributing to the regulation of mROS homeostasis and autophagic cell death.^33^ The role of SIRT3 in mediating the response to cardiac I/R injury was examined in our study. Here, we demonstrated that I/R reduced SIRT3 protein expression and activity, thus regulating the transcriptional activity of SOD2 by modulating acetylation. In the present study, the loss of SIRT3 did not additionally augment myocardial infarct size or worsen cardiac systolic function in 8‐week‐old mice exposed to I/R injury, which was consistent with the finding that 10‐week‐old SIRT3‐deficient mice did not show worsened cardiac function or expanded myocardial infarct sizes than wild‐type mice exposed to I/R injury.^34^ In contrast, 7‐month‐old SIRT3 heterozygous mice were developed worsened cardiac function and increased infarct sizes following I/R injury.^35^ We hypothesized that the age‐related change in the heart helped SIRT3‐deficient mice withstand I/R injury, while these mechanisms were less prominent in heterozygous mice. In addition, the reasons for these distinctly different results may be attributed to the different contributions of SIRT3 to autophagy during I/R with and without hypertrophic preconditioning.

Hypertrophic preconditioning increased SIRT3 activity (Figure 2A), promoted cardiomyocyte survival (Figure 1A,B) and improved cardiac function (Figure 1C) in WT mice following I/R injury, whereas SIRT3 deficiency reversed the aforementioned effects of hypertrophic preconditioning on the heart by regulating the SOD2‐mROS‐autophagy pathway (Figures 3,4). Importantly, adenovirus‐mediated myocardial SIRT3 overexpression mitigated SOD2 acetylation, reduced mROS and protected against autophagic cell death (Figure 5), suggesting that SIRT3 activation at least partially mimics the effects of hypertrophic preconditioning during I/R injury. Collectively, these results suggest that SIRT3 plays a pivotal role in the protective effects of hypertrophic preconditioning.

The upregulation of autophagy was shown to be cardioprotective during myocardial ischaemia, whereas the myocardial effects of autophagy during reperfusion are still unclear.^31^, ^36^, ^37^ Increased autophagy during reperfusion has been shown to be detrimental.^24^, ^36^ These findings are consistent with our results demonstrating that destructive autophagosome processing during I/R was associated with worsened cardiac function and expanded infarct size, while TAC preconditioning‐mediated autophagy inhibition was cardioprotective (Figures 1,2). However, it was also noted that the upregulation of autophagy during reperfusion conferred myocardial protective effects.^38^, ^39^ The reasons for these distinctly different results are incompletely understood but may be attributed to differences in I/R models, cell types and animal species. For example, autophagy stimulation is protective in HL‐1 cells exposed to simulated I/R injury or in mice that received limb ischaemic post‐conditioning.^38^, ^39^ We hypothesized that autophagy could induce differential downstream pathways under distinct experimental conditions. Our results that hypertrophic preconditioning inhibited mitochondrial‐derived superoxide production and blocked autophagic cell death have not yet been fully clarified.

In addition to the direct regulation of the SIRT3 pathway in I/R‐induced hearts, the protective effects of TAC preconditioning were mediated by reducing autophagic cell death in I/R‐induced mouse hearts in vivo. This finding was demonstrated by the following evidence: (1) TAC preconditioning ameliorated the dysfunctional autophagosome processing in the I/R‐induced myocardium (Figures 7,8); (2) TAC preconditioning reduced I/R‐induced Beclin 1 overexpression (Figure 2E); and (3) Beclin 1 knockdown mitigated I/R‐induced autophagy dysfunction and restored autophagic flux without an additive effect with the protection of TAC preconditioning, while Beclin 1 overexpression abolished the aforementioned protective effects of TAC preconditioning (Figure 7B). The findings of the present study that myocardial I/R‐induced autophagy and impaired autophagic flux (Figure 2D,F) are consistent with those of previous studies,^27^, ^36^ although the potential mechanisms by which a variety of interventions regulate Beclin 1 expression need to be elucidated.

During I/R, Beclin 1 activation has been shown to elicit an autophagic response in the heart and promote cell death.^36^ Consistent with previous reports,^27^, ^36^ our study showed that I/R markedly induced Beclin 1 activation in intact hearts, and this effect was reversed by the retraction of pressure overload after 3 days. Beclin 1 knockdown by in vivo lentivirus infection inhibited autophagic cell death and salvaged myocardial infarction, while Beclin 1 overexpression abrogated the protective effect of hypertrophic preconditioning against myocardial infarction in I/R‐induced hearts. These findings are consistent with previous reports showing that Beclin 1‐deficient mice are resistant to myocardial injury via the attenuation of autophagy.^27^, ^36^ In addition, we demonstrated that Beclin 1 knockdown reduced the myocardial LC3‐II/LC3‐I ratio and P62 expression, while Beclin 1 overexpression blocked TAC preconditioning‐mediated autophagosome processing, as evidenced by the increased LC3‐II/LC3‐I ratio and P62 expression. Therefore, we conclude that the significant induction of Beclin 1 activation by myocardial I/R may lead to impaired autophagic flux, whereas the inhibition of Beclin 1 by TAC preconditioning during I/R may contribute to the maintenance of autophagic flux and protect against myocardial I/R injury. Further studies are needed to determine whether other effects are involved in TAC preconditioning‐mediated cardioprotection.

Our study has shown that TAC preconditioning reduced myocardial autophagic cell death in a SIRT3/SOD2 pathway‐dependent manner, and however, the underlying cardioprotective mechanisms of hypertrophic preconditioning are still largely unknown. Wei et al reported that S100a8/a9 was involved in the protective effects of hypertrophic preconditioning against cardiac hypertrophy in adult mice.^19^ Moreover, pre‐treatments with angiotensin II or norepinephrine activate protein kinase C and limit myocardial infarction in isolated perfused rabbit hearts, which indicates that protein kinase C may contribute to the protective effect of hypertrophic preconditioning in I/R hearts.^14^ Future studies are needed to investigate the potential endogenous protective mechanisms of hypertrophic preconditioning, particularly in cardiac I/R injury.

Intriguingly, none of the differences reported in the I/R group were changed by transient hypertrophic stimulation alone, indicating that the observed differences may be secondary rather than primary effects of hypertrophic preconditioning. The present study suggested that SIRT3 was essentially required for the development of myocardial hypertrophic preconditioning and may mediate this process. However, the endogenous regulators of SIRT3 in myocardial I/R injury, particularly in hypertrophic preconditioning, remain largely unknown. A recent study from Kong et al reported that PGC‐1α may serve as an upstream regulator of SIRT3 and induced murine SIRT3 activation in muscle cells.^40^ PIKfyve was shown to phosphorylate SIRT3 and prevent myocardial apoptosis and hypertrophy in obese mice.^41^ In addition, exogenous NAD supplementation was shown to promote SIRT3 activation and attenuate Ang‐II‐induced cardiac hypertrophy by maintaining intracellular NAD levels.^42^ Therefore, we hypothesized that the intracellular NAD/NADH ratio may contribute to SIRT3 activation in response to hypertrophic preconditioning. Further studies are warranted to determine the endogenous regulators of SIRT3 in myocardial I/R injury, particularly in the context of hypertrophic preconditioning‐induced myocardial protection.

In summary, we identified the mechanism by which I/R‐induced cardiomyocyte death *via* mROS‐dependent autophagy. Importantly, we reported that hypertrophic preconditioning exerted marked protective effects by eliminating mitochondrial‐derived superoxide and suppressing autophagic cell death *via* the SIRT3‐SOD2 pathway. Our findings provide new insights into the link between hypertrophic preconditioning and autophagy signalling. Revealing the underlying mechanisms of hypertrophic preconditioning‐induced myocardial protection may help us to identify novel therapeutic targets to prevent I/R damage and slow the progression of heart failure.

## CONFLICT OF INTEREST

None.

## AUTHOR CONTRIBUTIONS

Lei‐Lei Ma, Fei‐Juan Kong, Kai‐Yue Xin, Zheng Dong, Ai‐Jun Sun, Yun‐Zeng Zou and Jun‐Bo Ge contributed to the initial experimental discussion and designs. Lei‐Lei Ma and Fei‐Juan Kong set up the animal model, analysed the data and wrote the manuscript. Lei‐Lei Ma, Xing‐Xu Wang, Kai‐Yue Xin, Zheng Dong and Ai‐Jun Sun performed experiments. Yun‐Zeng Zou and Jun‐Bo Ge wrote or revised the manuscript. All authors have reviewed the final manuscript and approved the submission to this journal.

## Supporting information

Fig S1‐S3

## Data Availability

All relevant data are available from the authors upon reasonable request.
