# Supplementary material for: Hypertrophic Preconditioning Attenuates Myocardial Ischaemia‐Reperfusion Injury by Modulating SIRT3‐SOD2‐mROS‐Dependent Autophagy
Source: Cell Prolif. 2021 May 11;54(7):e13051. doi: 10.1111/cpr.13051 (PMC8249780; doi:10.1111/cpr.13051)
Supplement: Supplementary file 1 — Fig S1‐S3 [file CPR-54-e13051-s001.doc]

**Supplemental Figure 1.**

**
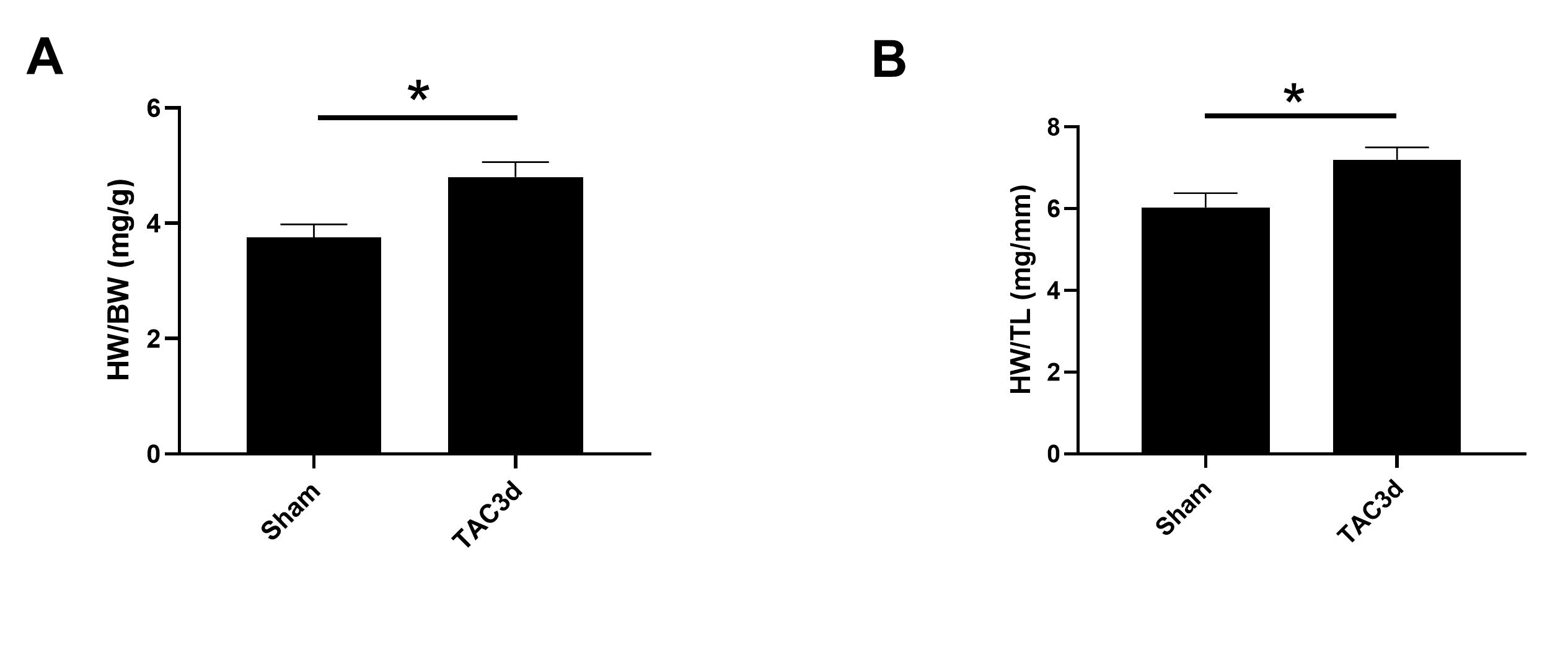
**

**Supplemental Figure 1. Cardiac hypertrophy induced by TAC for 3 days.**

The heart weight/body weight ratio (HW/BW) (**Supplemental Figure 1A**) and heart weight/tibial length ratio (HW/TL) (**Supplemental Figure 1B**) were significantly increased in response to TAC for 3 days. **P* < 0.05 compared with the Sham group; N = 6 mice per group.

**Supplemental Figure 2.**

**
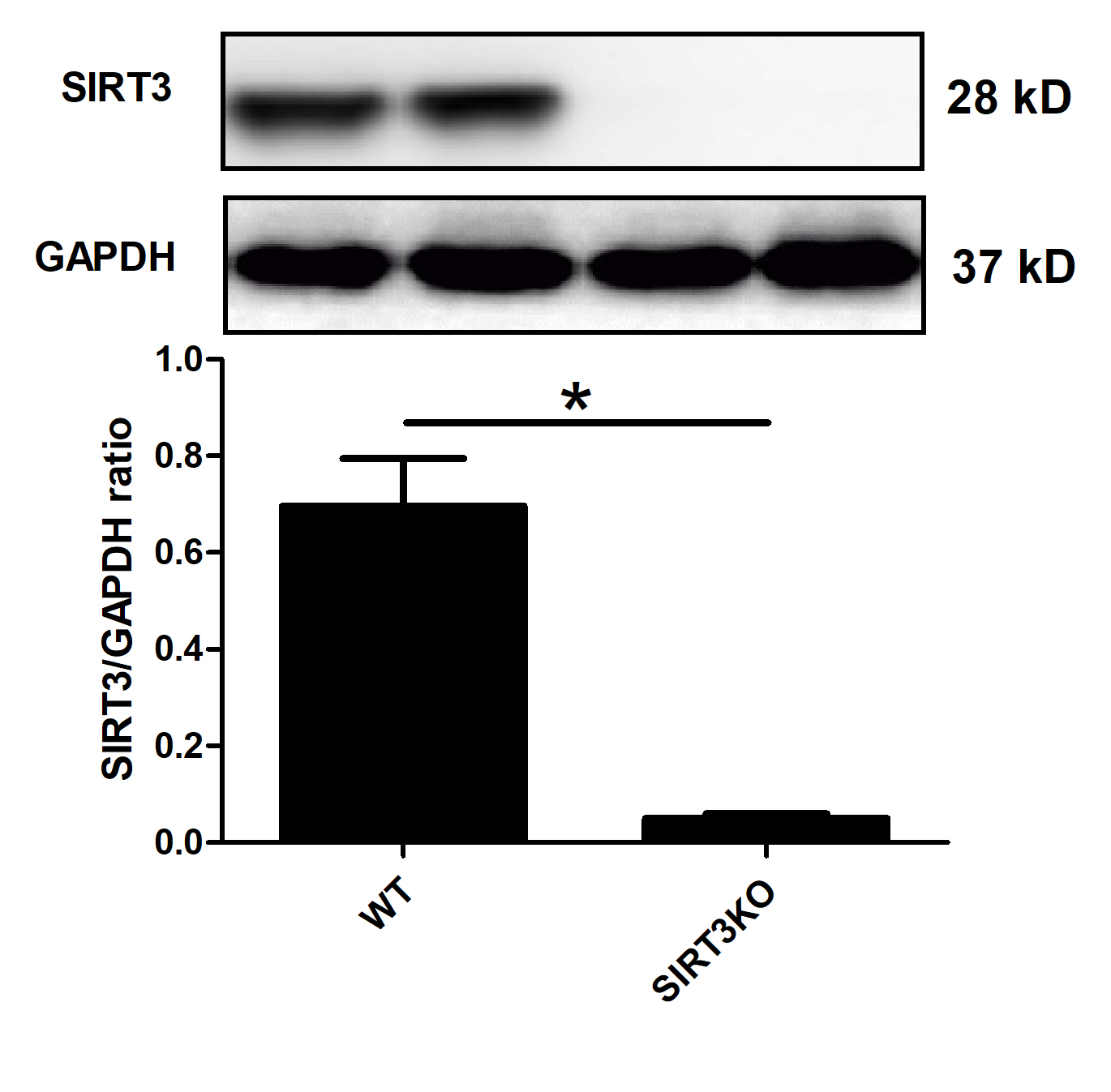
**

Supplemental Figure 2. Myocardial SIRT3 protein expression in wild type and SIRT3 knockout mice. n=6 mice per group, ^*^*P* < 0.05.

**Supplemental Figure 3.**


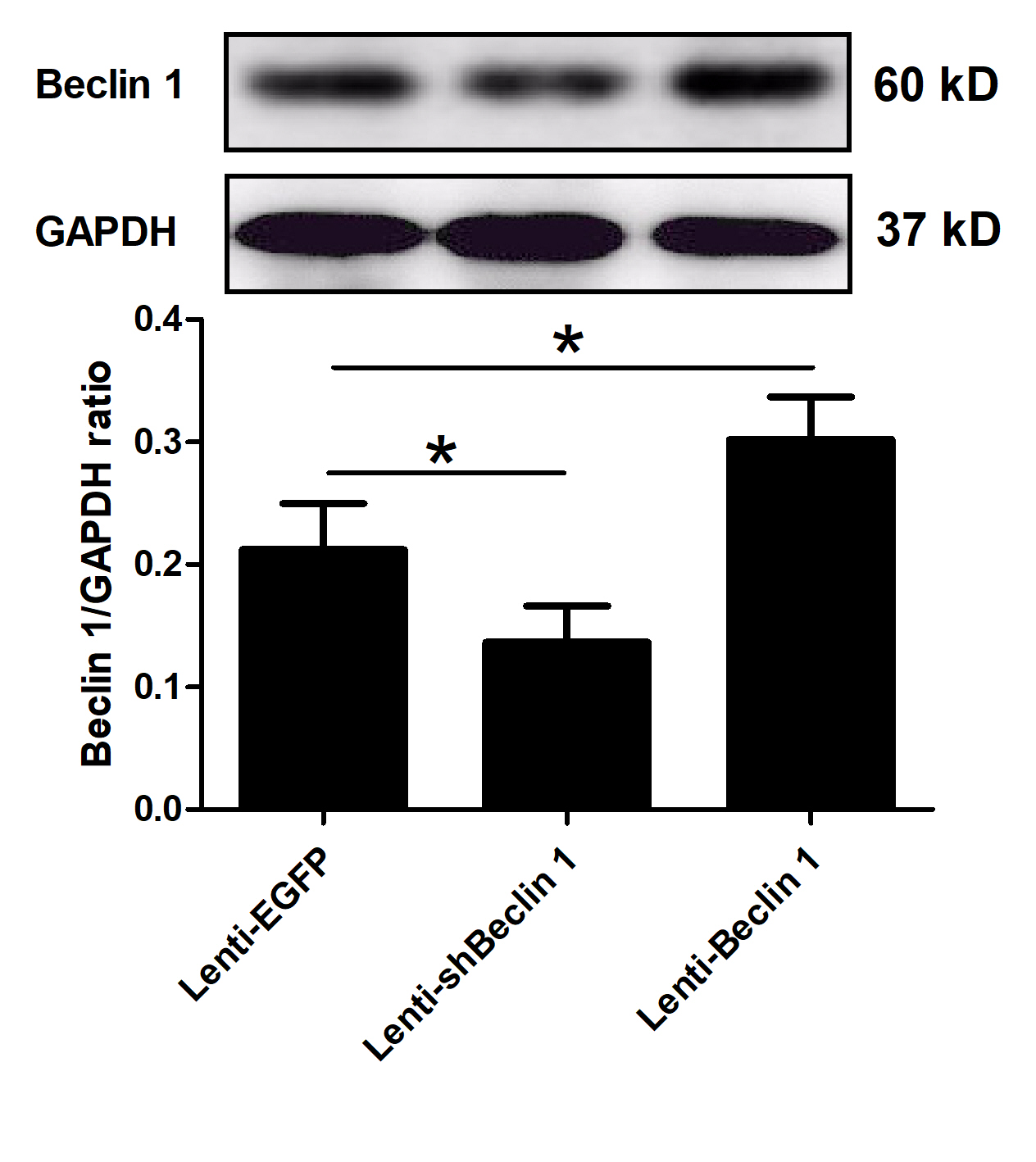


Supplemental Figure 3. Myocardial Beclin 1 protein expression in the mice heart infected by Lenti-EGFP, Lenti-shBeclin 1 or Lenti-Beclin 1. n=6 mice per group, ^*^*P* < 0.05.
